# Relationship of workplace violence and perpetrators on sleep disturbance-data from the 4th Korean working conditions survey

**DOI:** 10.1186/s40557-016-0142-z

**Published:** 2016-10-19

**Authors:** Taejun Yoo, Byeongjin Ye, Jung-Il Kim, Siwoo Park

**Affiliations:** Department of Occupational & Environmental Medicine, College of Medicine, Dong-A University, Busan, South Korea

**Keywords:** Workplace violence, Sleep disturbance, Korean working conditions survey

## Abstract

**Objective:**

The present study analyzed relationship of workplace violence and perpetrators of violence on sleep disturbance among wage workers in Korea.

**Methods:**

The present study used data from the 4th Korean Working Conditions Survey (KWCS) of 2014 in selecting a total of 25,138wage workers as the study population, which excluded those who failed or refused to respond to questions required for the present study. The workplace violence experience group included people who satisfied at least one of six relevant criteria (verbal abuse, unwanted sexual attention, threatening or humiliating behavior, physical violence, bullying/harassment, and sexual harassment) and the group was divided according to whether the perpetrator of violence was a client or colleague. Presence of sleep disturbance was determined based on subjective symptoms felt within the past 12 months by each individual. A multiple logistic regression analysis was performed to identify the effects on sleep distance according to general, occupational, and psychosocial characteristics, as well as the types of workplace violence and perpetrators of violence.

**Results:**

Workplace violence was found as a factor affecting sleep disturbance (OR = 3.773, 95 % CI = 3.058–4.655), and with respect to perpetrators of violence, complaint of sleep disturbance symptoms was higher when the perpetrator was a colleague or boss (OR = 5.688, 95 % CI 4.189–7.723) than a client (OR = 2.992, 95 % CI 2.301–3.890).

**Conclusion:**

Workplace violence had an effect on occurrence of sleep disturbance and when the perpetrators of violence was a boss or colleague at work, the risk for symptoms such as sleep disturbance increased, which indicated the need for appropriate intervention from a workplace healthcare perspective, including preventive education of workplace violence among employees.

## Background

Violence can be defined as all forms of aggressive behavior intended to harm another person, which can include both psychological and physical aspects [1]. Meanwhile, workplace violence can be defined as being violated, threatened, harmed, or injured by certain behavior, event, or action pertaining to work outcome or rightful performance of work [2]. Various words are used, interchangeably, to define aggressive behavior in the workplace, and depending on the severity and frequency of the behavior, words such as aggression, violence, harassment, mistreatment, ostracism, incivility, and bullying or mobbing can be used [3].

Workplace violence is closely associated with mental health of the workers, and is a serious problem which can have a negative effect on individual workers [4]. Victims of workplace violence can manifest symptoms of somatization, depression, anxiety, and negative affectivness and even witnesses of workplace violence have been reported to show symptoms such as anxiety [5]. The risk of poor subjective health, sickness absence, and presenteeism was high in the group which had experienced workplace violence and discrimination [6]. Moreover, workplace violence causes psychological exhaustion and post-traumatic stress syndrome [7], while also having a negative effect on the worker’s satisfaction and turnover intention [8].

Workplace violence not only causes mental health problems, but also decreases in the company’s productivity, and after workplace violence occurs, significant costs, such as medical costs, are incurred [9, 10].

Sleep is one of the basic human desires and is an important factor in maintaining health and quality of life. Sleep disturbance can cause symptoms of diminished mental function, such as vertigo, fatigue and instability, attention deficit disorder, sensory disorder, and increased aggression, while also being the cause of decreased productivity, industrial accidents, and traffic accidents [11]. Moreover, sleep disturbance is associated with heart disease, diabetes, and metabolic disorders and sleep problems are known to be risk factors of these diseases [12].

In the most recent 10 years, there have been many studies in Korea on workplace violence and overall health impact, but most of the studies examined specific occupations, such as medical and service industries, and studies on topic of associations with sleep disturbance have been hard to find [13–16]. In particular, studies on the effects of perpetrators of violence on sleep disturbance are almost non-existent.

Accordingly, the present study used the 4^th^KWCS data, representative of wage workers in Korea, to identify the effects of workplace violence and perpetrator of violence on complaints of sleep disturbance symptoms and to analyze the associations between workplace violence and occupational factors.

## Methods

### Subjects

The present study used data from the 4th KWCS, which was conducted between June and September 2014 on employed workers, 15 years or older. Among a total of 50,007 people, 25,138 wage workers were selected as the subjects of the study, after excluding military personnel and those who failed to or refused to respond to the questions required for analyses.

### General characteristics

General characteristics included sex, age, education level, and income.

### Occupational characteristics

With respect to occupational characteristics, job types were divided into blue collar (skilled agricultural and fishery workers, craft and related trades workers, plant and machine operators and assemblers, and elementary occupations), service work (service workers and sales workers), and white collar (manager, professional, technicians and associate professionals, and office workers). Occupational characteristics also included type of employment, number of employees, working time, shift work, and emotional labor. Emotional labor was defined if the response to the question “In my work, I have to hide my feelings” was either”always” or “most of the time”. Being exposed to physical risk factors (vibration, noise, high and low temperatures), biochemical risk factors (dust, organic solvents, chemical handling, second hand smoke, and infectious materials handling and contact), and ergonomic risk factors (posture that cause fatigue or pain, lifting or moving people, heavy load handling, continuous standing posture, and repetitive movement) was defined as exposure for ≥1/4 of working time.

Psychosocial risk factors were divided into 5 categories of job demand, job control, social support, job insecurity, and lack of reward. Job demand used 7 questions, which consisted of; “very fast speed,” “strict deadline,” and “experience of stopping task at hand for unexpected work” for work speed; “strict product quality standards,” “evaluating one’s own quality of work,” and “resolve unexpected problems on one’s own”for occupational characteristics; and “enough time to complete the task at hand” for work situation. Job control used 7 questions, which consisted of “possible to take care of personal business during work hours,” along with “order of work,” “work method,” and “work speed/work rate” for possibility of work selection and “I have a say in the choice of my working partners,” “I can take a break when I wish,” and “I can influence decisions that are important for my work” for work situation. Social support used 8 questions, which consisted of “support of colleagues,” “support of boss,” and “I have a very good friend at work” for job situations and “gives feedback to my work,” “respects me as an individual,” “resolves conflicts well,” “plans and organizes work very well,” and “encourages me to participate in important decision making” for disposition and attitude of direct superior. Job insecurity used only 2 questions, which were “I will lose this job within the next 6 months” and “Even if I quit or get fired from this job, it will be easy for me to find another job with similar pay.” Lack of reward only used 1 question, which was “I am receiving fair compensation for my work.” Each question was scored and the subjects were divided into high or low group according to the median score. With respect to groups, score of <3 points was considered low job demand group and score of ≥4 was considered high job demand group; <3 points was considered low insufficient job control group and score of ≥4 was considered high insufficient job control group; <1 point was considered low inadequate social support group and score of ≥2 was considered high inadequate social support group; and 0 point was considered low job insecurity group and score of ≥1 was considered high job insecurity group. Since lack of reward contained only 1 question, inadequate compensation was given a score of 1 point. The internal consistency of each of the psychosocial factors is as follows. The Cronbach alpha value was 0.630 for job demand (7 questions), 0.670 for job autonomy (7 questions), and 0.793 for social support (8 questions).

### Workplace violence and perpetrators of violence

Workplace violence was assessed using the following 6 questions; “In the past 1 month, have you experienced the following?” A) verbal abuse, B) unwanted sexual attention, or C) threatening or humiliating behavior?” and “In the past 1 year, have you experienced the following? A) physical violence, B) bullying/harassment, or C) sexual harassment?” Six types of workplace violence were measured each with Yes/No response, No opinion/Refusal categories. If one or more items were answered “Yes”, the respondent was identified as a member of workplace violence experience group.. The workplace violence experience group was divided based on whether their response to the question on perpetrator of violence was a client (client, student, patient, etc.) and/or colleague (boss, colleague, subordinate, etc.).

### Sleep disturbance

Anyone who answered “Yes” to sleep disturbance or insomnia for the question “Have you had any of the following health problems in the past 12 month” was considered as having sleep disturbance. KWCS includes a survey item regarding sleep disorder symptomswhich considers a job-related problem as a cause. However, in the current study, the survey item regarding sleep disorder symptoms irrelevant to job was used as the dependent variable because we hypothesized that sleep disorder may be caused by workplace violencenot recognized as directly related to problems at work.

### Analysis method

In order to examine the associations of general characteristics, occupational characteristics, workplace violence, and perpetrators of violence with sleep disturbance, a chi-squared test was performed. Based on the factors which showed statistical significance in the chi-squared test results, a multiple logistic regression analysis was used to examine the effects of general characteristics, occupational characteristics, workplace violence, and perpetrators of violence on symptoms of sleep disturbance. All analyses were performed using SPSSver. 22.0 (Chicago, IL, USA).

## Results

### Subject characteristics

There were slightly more males than females and those with age of 30 to 40 years account for 56.6 % of all subjects. Highest distribution for education level and income was shown as graduation of college or above and 1 to 2 million KRW, respectively. Distribution by job type showed a descending order of service work, blue collar, and white collar, while the highest distribution was shown as regular workers for type of employment; 5–49 employees for number of employees; and ≤40 hr for working time. The percentage of subjects who work shifts and engage in emotional labor was 9.8 and 25.8 %, respectively, while the percentage of subjects who had physical, biochemical, and ergonomic risk factors was 37.6, 22.5, and 81.0 %, respectively. With respect to psychosocial factors, there were slightly more subjects in the groups with high job demand, insufficient job control, and inadequate social support, while the group with low job insecurity and the group with fair compensation were shown to be significantly higher.. In the items for workplace violence, a total of 1,510 people had experienced workplace violence and there were more cases of the perpetrator of violence being a client rather than a colleague or a boss (Table 1).

**Table 1:** Subject characteristics (2014 KWCS)

Characteristics	Classification	N (%)
Sex	Male	13214 (52.6 %)
	Female	11924 (47.4 %)
Age	<30	3516 (14.0 %)
	30–39	6856 (27.3 %)
	40–49	7365 (29.3 %)
	50–59	4831 (19.2 %)
	≥60	2570 (10.2 %)
Education	Middle school or below	2530 (10.1 %)
	High school	8981 (35.7 %)
	College or above	13626 (54.2 %)
Income (10 thousands)	<100	2555 (10.2 %)
	100–199	8526 (33.9 %)
	200–299	7601 (30.2 %)
	≥300	6456 (25.7 %)
Job type	Blue collar	7499 (29.8 %)
	Service work	5825 (23.2 %)
	White collar	11814 (47.0 %)
Type of employment	Regular	19734 (78.5 %)
	Temporary	5404 (21.5 %)
Number of employees	<5	4556 (18.1 %)
	5–49	13374 (53.2 %)
	50–299	4965 (19.7 %)
	≥300	2243 (8.9 %)
Working time	≤40	13240 (52.7 %)
	41–52	7552 (30.0 %)
	53–60	3093 (12.3 %)
	≥61	1253 (5.0 %)
Shift work	No	22682 (90.2 %)
	Yes	2456 (9.8 %)
Emotional labor	No	18654 (74.2 %)
	Yes	6483 (25.8 %)
Physical risk factors	No	15684 (62.4 %)
	Yes	9454 (37.6 %)
Biochemical risk factors	No	19486 (77.5 %)
	Yes	5652 (22.5 %)
Ergonomic risk factors	No	4785 (19.0 %)
	Yes	20353 (81.0 %)
Job demand	Low	15625 (62.2 %)
	High	9513 (37.8 %)
Insufficient job control	Low	13546 (53.9 %)
	High	11592 (46.1 %)
Inadequate social support	Low	15605 (62.1 %)
	High	9533 (37.9 %)
Job insecurity	Low	15726 (62.6 %)
	High	9412 (37.4 %)
Lack of reward	Low	19730 (78.5 %)
	High	5408 (21.5 %)
Workplace Violence	No	23627 (94.0 %)
	Yes	1510 (6.0 %)
Perpetrators	None	23627 (94.0 %)
	Client	1052 (4.2 %)
	Coleague	459 (1.8 %)

### Associations of general characteristics, occupational characteristics, and workplace violence with respect to sleep disturbance

The factors associated with sleep disturbance symptoms were sex, age, number of employees, working time, shift work, emotional labor, ergonomic risk factors, job demand, job control, social support, job insecurity, lack of reward, workplace violence, and perpetrators of violence (Table 2).

**Table 2:** Associations of general characteristics, occupational characteristics, and workplace violence with respect to sleep disturbance

		Sleep disturbance						
		No	%	Yes	%	Total	%	*p*-value^a^
	Total	24536	97.6 %	602	2.4 %	25138	100.0 %	
Sex	Male	12931	97.9 %	282	2.1 %	13214	52.6 %	0.004
	Female	11604	97.3 %	320	2.7 %	11924	47.4 %	
Age	<30	3424	97.4 %	92	2.6 %	3516	14.0 %	0.037
	30–39	6723	98.1 %	133	1.9 %	6856	27.3 %	
	40–49	7170	97.4 %	194	2.6 %	7365	29.3 %	
	50–59	4718	97.7 %	112	2.3 %	4831	19.2 %	
	≥60	2500	97.2 %	71	2.8 %	2570	10.2 %	
Education	Middle school or below	2460	97.2 %	71	2.8 %	2530	10.1 %	0.226
	High school	8781	97.8 %	200	2.2 %	8981	35.7 %	
	College or above	13295	97.6 %	331	2.4 %	13626	54.2 %	
Income (10 thousands)	<100	2489	97.4 %	66	2.6 %	2555	10.2 %	0.435
	100–199	8326	97.6 %	201	2.4 %	8526	33.9 %	
	200–299	7433	97.8 %	168	2.2 %	7601	30.2 %	
	≥300	6288	97.4 %	168	2.6 %	6456	25.7 %	
Job type	Blue collar	7334	97.8 %	165	2.2 %	7499	29.8 %	0.320
	Service	5687	97.6 %	138	2.4 %	5825	23.2 %	
	White collar	11514	97.5 %	300	2.5 %	11814	47.0 %	
Type of employment	Regular	19251	97.6 %	483	2.4 %	19734	78.5 %	0.296
	Temporary	5285	97.8 %	119	2.2 %	5404	21.5 %	
Number of employees	<5	4475	98.2 %	81	1.8 %	4556	18.1 %	0.001
	5–49	13060	97.7 %	314	2.3 %	13374	53.2 %	
	50–299	4817	97.0 %	148	3.0 %	4965	19.7 %	
	≥300	2183	97.3 %	60	2.7 %	2243	8.9 %	
Working time	≤40	12973	98.0 %	267	2.0 %	13240	52.7 %	<0.001
	41–52	7342	97.2 %	210	2.8 %	7552	30.0 %	
	53–60	3029	97.9 %	64	2.1 %	3093	12.3 %	
	≥61	1192	95.1 %	61	4.9 %	1253	5.0 %	
Shift work	No	22180	97.8 %	501	2.2 %	22682	90.2 %	<0.001
	Yes	2355	95.9 %	101	4.1 %	2456	9.8 %	
Emotional labor	No	18267	97.9 %	387	2.1 %	18654	74.2 %	<0.001
	Yes	6268	96.7 %	215	3.3 %	6483	25.8 %	
Physical risk factors	No	15311	97.6 %	373	2.4 %	15684	62.4 %	0.824
	Yes	9224	97.6 %	229	2.4 %	9454	37.6 %	
Biochemical risk factors	No	19029	97.7 %	457	2.3 %	19486	77.5 %	0.340
	Yes	5507	97.4 %	145	2.6 %	5652	22.5 %	
Ergonomic risk factors	No	4706	98.3 %	79	1.7 %	4785	19.0 %	<0.001
	Yes	19830	97.4 %	523	2.6 %	20353	81.0 %	
Job demand	Low	15296	97.9 %	329	2.1 %	15625	62.2 %	<0.001
	High	9240	97.1 %	273	2.9 %	9513	37.8 %	
Insufficient job control	Low	13246	97.8 %	300	2.2 %	13546	53.9 %	0.043
	High	11290	97.4 %	302	2.6 %	11592	46.1 %	
Inadequate social support	Low	15329	98.2 %	276	1.8 %	15605	62.1 %	<0.001
	High	9207	96.6 %	326	3.4 %	9533	37.9 %	
Job insecurity	Low	15432	98.1 %	294	1.9 %	15726	62.6 %	<0.001
	High	9103	96.7 %	308	3.3 %	9412	37.4 %	
Lack of reward	Low	19278	97.7 %	452	2.3 %	19730	78.5 %	0.040
	High	5258	97.2 %	150	2.8 %	5408	21.5 %	
Workplace Violence	No	23158	98.0 %	469	2.0 %	23627	94.0	<0.001
	Yes	1377	91.2 %	133	8.8 %	1510	6.0	
Perpetrators	None	23158	98.0 %	469	2.0 %	23627	94.0 %	<0.001
	Client	977	92.9 %	74	7.1 %	1052	4.2 %	
	Coleague	400	87.2 %	59	12.8 %	459	1.8 %	

^a^Chi-Square test

### Association between general characteristics and sleep disturbance

It was found that in terms of sex, rate of sleep disturbance symptoms was higher among females than males.

### Association between occupational characteristics and sleep disturbance

Compared to number of employees totaling <5, the risk of sleep disturbance increased as the number of employees increased, with number of employees totaling ≥300 having the highest risk of sleep disturbance. Compared to working time of ≤40 hr, the risk of sleep disturbance was higher when working 41–52 and ≥61 hr. The risk of sleep disturbance was higher for engaging in shift work and emotional labor, as well as having ergonomic risk factors.

With respect to psychosocial factors, the risk of sleep disturbance was higher in the group with high job demand than low job demand; low social support than high social support; and high job insecurity than low job insecurity.

### Associations of workplace violence and perpetrators of violence with respect to sleep disturbance

Compared to other variables, workplace violence had the largest effect on sleep disturbance.(OR = 3.779, 95 % CI = 3.064–4.650). The risk of sleep disturbance was higher when the perpetrator of violence was a boss or a colleague (OR = 5.684, 95 % CI 4.195–7.702) than when it was a client (OR = 2.991, 95 % CI 2.301–3.889) (Table 3).

**Table 3:** Effects on sleep disturbance according to general characteristics, occupational characteristics, workplace violence, and perpetrators of violence

Variable	OR^a^	95 % CI	OR^b^	95 % CI
Sex (ref. Male)	1.388	1.168–1.648	1.408	1.185–1.673
Age	1.001	0.994–1.008	1.001	0.995–1.008
Number of employees (ref. < 5)				
5–49	1.484	1.153–1.910	1.469	1.141–1.890
50–299	1.984	1.494–2.637	1.956	1.472–2.600
≥300	2.091	1.463–2.987	2.039	1.426–2.916
Working time (ref. ≤ 40)				
41–52	1.332	1.103–1.607	1.328	1.100–1.603
53–60	0.955	0.718–1.271	0.954	0.716–1.270
≥61	2.169	1.595–2.951	2.178	1.601–2.963
Shift work (ref. No)	1.425	1.128–1.799	1.488	1.177–1.881
Emotional labor (ref. No)	1.495	1.256–1.780	1.509	1.268–1.797
Ergonomic risk factors (ref. No)	1.287	1.005–1.649	1.310	1.022–1.679
Job demand (ref. Low)	1.298	1.095–1.539	1.261	1.062–1.497
Insufficient job control (ref. Low)	0.916	0.771–1.087	0.908	0.765–1.078
Inadequate social support (ref. Low)	1.838	1.542–2.191	1.812	1.520–2.161
Job insecurity (ref. Low)	1.723	1.454–2.041	1.718	1.450–2.035
Lack of reward (ref. Low)	0.885	0.726–1.078	0.863	0.707–1.053
Workplace Violence (ref. No)	3.779	3.064–4.650		
Perpetrators (ref. None)				
Client			2.991	2.301–3.889
Colleague or boss			5.684	4.195–7.702

^a^ Multiple logistic regression analysis including variables of sex, age, number of employees, working time, shift work, emotional labor, ergonomic risk factors, job demand, job control, social support, job insecurity, lack of reward, and workplace violence

^b^Multiple logistic regression analysis including variables of sex, age, number of employees, working time, shift work, emotional labor, ergonomic risk factors, job demand, job control, social support, job insecurity, lack of reward, and perpetrators of violence

## Discussion

The present study used the 4^th^KWCS in analyzing workplace violence and sleep disturbance of wage workers in Korea. The risk of sleep disturbance appears higher in females than in males, which was consistent with other study results showing higher prevalence of sleep disturbance in females than in males [17–19]. The risk of sleep disturbance was higher in those who engage in shift work [20–22], engage in emotional labor [23], and work long hours [24], which was consistent with previous study results as well.

Among the psychosocial risk factors, prevalence of sleep disturbance was higher when job demand was higher and social support was lower, which was consistent with precedent study which reported that high job demand and low social support increased prevalence of sleep disturbance [21, 25]. In a study by Kim et al. [26], factors such as job control, job insecurity, and lack of reward showed statistically significant results with sleep disturbance, but such results varied depending on the study [25, 27, 28].

The risk of sleep disturbance was much higher in the group that experienced workplace violence than the group that did not, which was similar to foreign studies that showed higher risk of sleep disturbance in the group exposed to workplace bullying [29, 30].

With respect to the mechanism by which workplace violence causes sleep disturbance, it has been reported that occupational stress, such as workplace violence, can directly cause sleep disturbance through cortisol and hypothalamic-pituitary-adrenal axis activation [31, 32]. However, according to other study results, workplace violence causes fear-mediated turnover intentions and physical symptoms in the victims [33], which is similar to a study case that reported workplace bullying caused sleep disturbance mediated by negative affectivity, need for recovery, and worry [34]. Moreover, sleep disturbance has a bidirectional relationship with depression in that it is an outcome of depression as well as a risk factor of depression [35] and considering the fact that workplace violence is significantly correlated with depression [4], workplace violence can affect manifestation of sleep disturbance symptoms in people who experienced workplace violence, mediated by negative emotions, such as depression or anxiety.

One of the results that is worth noting in the present study was the fact that the odds ratio of sleep disturbance symptom was much higher when the perpetrator of violence was a colleague or a boss (OR = 5.684, 95 % CI = 4.195–7.702) rather than a client (OR = 2.991, 95 % CI = 2.301–3.889). It is believed that these results are attributable to conflict with a boss or colleague being a major source of job stress [26]. This is similar to the results from a study by Byun which indicated that relationships between colleagues, such as ignoring and bullying have a bigger influence on expression of depressive symptoms than simple verbal abuse [13]. As consideration of another reason for these results, it can be surmised that the reason why violence perpetrated by a boss or colleague has the characteristics of being more continuous and repetitive than that by a client. According the definition given by Leymann [36],workplace bullying is defined as behavior that continues for 6 or more months and repeats more than once a week, and unlike violence perpetrated by a client, workplace violence, such as verbal abuse, ignoring, or threat perpetrated by a colleague or boss may also have repetitive characteristics rather than being a one-time event. As the frequency and severity of such violent behavior increase and the behavior persists longer, such behavior shows a pattern of becoming more serious [37], and as such, the degree of manifestation of mental symptoms such as sleep disturbance or depression may also become more serious according to the frequency and seriousness of the behavior. A study by NIedhammer [30] also showed that as workplace bullying became more frequent, the risk of sleep disturbance symptoms also increased.

The present study had few limitations. First, KWCS is a cross-sectional survey. Also We classified the respondents into the workplace violence group, if they had any one of the experiences of verbal abuse,unwanted sexual attention, and threatening or humiliating behaviorwithin the past 1 month, and physical violence, bullying/harassment, and sexual harassmentwithin the past 1 year. KWCS are designed with two time periods according to workplace violence type. Because we used the KWCS data, we could not avoid the limitation by using these two time periods in the operational definition of workplace violence. Due to cross-sectional study and the difference in time period, we were not able to determine the direct causal relationship between sleep disorder and workplace violence.. However, considering that a precedent study reported that job stress was a cause of sleep disorders, such as insomnia and sleep disturbance [38] and looking at the mechanism by which workplace violence causes sleep disturbance, it would be valid to believe that workplace violence is a cause of sleep disturbance [31–34].

Second, workplace violence experience or presence of sleep disturbance symptoms was measured according to subjective reporting by the subject. Therefore, there are limitations in the validity and reliability of the measurement tools.

Third, causes of sleep disturbance include alcohol consumption, caffeine consumption, drug use and abuse, mental illness, and respiratory diseases [39],but since these factors were not investigated, their effects are unknown.

Despite these limitations, the present study used data from 2014 KWCS, data representative of Korea, in identifying significant association between workplace violence and sleep disturbance of wage workers in Korea. Moreover, the study also found that the risk of sleep disturbance symptoms was higher when the perpetrator of violence was a colleague or a boss.

## Conclusion

Workplace violence had a significant effect on sleep disturbance, and in particular, the odds of sleep disturbance were much higher when the perpetrator of the violence was a colleague or a boss. Therefore, it would be necessary to conduct proper intervention, such as workplace violence education, in order to prevent workplace violence between colleagues.
